# Benchmarking major somatic structural variant callers on the HG008 genome

**DOI:** 10.3389/fgene.2026.1732039

**Published:** 2026-05-12

**Authors:** Xinran Cui, Yadong Liu, Long Qian, Yadong Wang

**Affiliations:** 1 School of Medicine and Health, Faculty of Life Sciences and Medicine, Harbin Institute of Technology, Harbin, Heilongjiang, China; 2 Center for Bioinformatics, Faculty of Computing, Harbin Institute of Technology, Harbin, Heilongjiang, China; 3 Key Laboratory of Biological Bigdata, Ministry of Education, Harbin Institute of Technology, Harbin, Heilongjiang, China; 4 Center for Quantitative Biology, Academy for Advanced Interdisciplinary Studies, Peking University, Beijing, China

**Keywords:** benchmarking, cancer genomics, ensemble strategy, long-read sequencing, somatic structural variants, variant calling tools

## Abstract

Somatic structural variants (SVs) are the predominant source of cancer driver mutations and play a critical role in oncogenesis. Comprehensive characterization of somatic SVs is critical for elucidating the mechanisms underlying tumorigenesis and for identifying biomarkers with diagnostic and therapeutic potential. However, their accurate detection remains challenging, primarily because most existing SV detection algorithms were originally developed for germline variants and are not well-suited to addressing the high heterogeneity of somatic mutations. In recent years, although several tools specifically designed for somatic SVs have emerged, their detection performance has not yet been rigorously validated. To bridge this gap, we conducted a comprehensive benchmarking of four leading somatic SV detection tools, namely, Sniffles2, Nanomonsv, Savana, and Severus, on the HG008 genome from Genome in a Bottle Consortium (GIAB). Their outputs were evaluated against the HG008 clonal somatic SV draft benchmark to assess overall performance. We further integrated the somatic SV callsets from multiple tools and compared them with the benchmark set, thereby establishing a multi-tool ensemble strategy for SV detection to achieve more accurate and comprehensive identification of somatic SVs.

## 1 Introduction

Structural variants (SVs) are defined as genomic alterations exceeding 50 base pairs in length, including deletions, insertions, duplications, inversions, and related events (Cosenza et al., 2022). Broadly, SVs can be classified into two categories: germline SVs, which are inherited and present in all cells, and somatic SVs, which occur post-zygotically in proliferative tissues. In cancer, somatic SVs are particularly pervasive, representing key genomic alterations that drive tumor initiation, progression, and therapeutic resistance (ICGC/TCGA Pan-Cancer Analysis of Whole Genomes Consortium, 2020). The rapid advancement of long-read genomic sequencing technologies has created unprecedented opportunities to resolve large and complex somatic genetic alterations (Espinosa et al., 2024). However, the heterogeneity of cancer genomes often challenges the capabilities of conventional germline SV analysis tools, leading to increased false-positive and false-negative calls (Keskus et al., 2025). Currently, tools specifically designed for somatic SV analysis remain scarce. In this study, we systematically evaluated the performance of four mainstream tools for somatic SV detection—Sniffles2 (Smolka et al., 2024), Nanomonsv (Shiraishi et al., 2023), Savana (Elrick et al., 2025), and Severus (Keskus et al., 2025).

Although all four tools can detect somatic SVs, they employ distinct algorithmic strategies that confer unique advantages. Sniffles2 enhances its performance through the introduction of repeat-aware clustering, rapid consensus sequence generation, and coverage-adaptive filtering, which markedly improve detection accuracy and efficiency (Smolka et al., 2024). Owing to its broad generalizability, Sniffles2 is appropriate for SV detection across autosomes, mosaic genomes, and population-scale studies, with mosaic analysis playing a key role in resolving non-germline and somatic SVs.

Nanomonsv comprises two detection modules: the canonical SV module and the single break-end (BND) SV module (Shiraishi et al., 2023). The former module is effective in identifying typical SVs in tumor–normal paired long-read data, capable of recovering SVs detected in short-read data as well as uncovering novel ones previously undetectable by short-read data. In contrast, the latter module is designed to capture complex somatic SV signals, including those arising from centromeric regions or mediated by mobile elements.

Savana detects both somatic SVs and copy number variations (CNVs), enabling a broader characterization of somatic genomic alterations (Elrick et al., 2025). Its key innovation lies in the integration of machine learning techniques, which analyze multiple features, such as variant position and type, supporting read count and orientation, as well as sequencing depth, to precisely distinguish true variants from sequencing or alignment artifacts, thereby significantly reducing false-positive rates.

Severus utilizes a breakpoint graph-based approach, supporting the use of matched normal samples and accommodating unbalanced cancer karyotypes (Keskus et al., 2025). Leveraging its ability to resolve multi-breakpoint SV architectures and produce haplotype-specific calls, this approach is effective for the precise identification of complex somatic SVs in long-read cancer genome analyses.

All four tools were employed to conduct somatic SV calling on the gold-standard Genome in a Bottle Consortium (GIAB) HG008 genome dataset. The HG008 dataset includes sequencing reads of the pancreatic ductal adenocarcinoma cell line HG008-T and its matched normal cell line HG008-N, derived from surgically resected pancreatic cancer tissue along with matched normal tissue from the duodenum and pancreas (McDaniel et al., 2025). A multi-platform strategy that integrates 17 technologies, such as short-read sequencing, long-read sequencing, and single-cell whole-genome sequencing, was used to produce the most comprehensive tumor–normal paired genome dataset to date. In this study, we utilized the HG008 long-read sequencing data generated by PacBio Revio HiFi sequencing and ONT PromethION nanopore sequencing. Unlike the COLO829 cancer cell line used in previous benchmarks, HG008 provides a clearer clonal background with reduced heterogeneity, thereby enabling a more robust performance evaluation of somatic SV detection. Grounded in a quantitative evaluation of the SV callers, we established a streamlined ensemble-3 framework; through rigorous head-to-head comparison with existing ensemble strategies and systematic downstream characterization of the integrated callsets, we substantiate the robustness and biological coherence of this framework for somatic SV detection.

## 2 Methods

### 2.1 Data sources and reference genome

We downloaded four paired tumor–normal long-read sequencing datasets generated on ONT and PacBio platforms. ONT simplex reads were basecalled with dorado (version 0.3.4) and aligned to the GRCh38-GIABv3 reference genome using Minimap2 (version 2.26) (Li, 2021), while PacBio HiFi reads were aligned to the same reference with pbmm2 (version 1.12.0). Both datasets achieved consistently high mapping rates. All downstream SV analyses were conducted against the GRCh38-GIABv3 reference genome to ensure analytical consistency.

### 2.2 Somatic SV calling pipelines

Somatic SVs were detected directly from the downloaded pre-aligned BAM files using four state-of-the-art long-read SV callers: Sniffles2 (version 2.6.1), Nanomonsv (version 0.8.0), Savana (version 1.3.5), and Severus (version 1.5). All tools were run in tumor–normal paired mode where applicable. Notably, as Sniffles2 was not specifically optimized for somatic SV detection, three complementary strategies were employed: tumor-only calling, tumor-only calling with the*–mosaic* parameter enabled, and a tumor–normal subtraction approach, in which SVs were called separately in tumor and normal samples, followed by removal of variants present in the normal to obtain putative somatic SVs (Feng et al., 2025). It should be clarified that the tumor-only mode refers to variant calling performed solely on the tumor sample, where most detected SVs are expected to be germline rather than somatic. The subtraction strategy addresses this limitation by filtering out variants shared with the matched normal sample.

SV analyses were executed with default parameters across all tools; consequently, default settings were applied to options such as minimum supporting reads and minimum SV length. However, since Savana employs different models for ONT and PacBio data, the*–ont* or*–pb* option was specified accordingly during execution. All analyses were performed in a high-performance computing environment, with details including an Intel Xeon Gold 6240 processor and a per-job resource allocation of 32 CPU cores and 120 GB of RAM, with software dependencies managed through Conda configurations.

To further quantify parameter robustness, we performed systematic sensitivity analyses to examine the influence of key settings on tool performance. For Sniffles2, the *-mosaic-af-max* parameter was tested at 0.5, 0.6, and 0.7. To ensure a fair comparison, three core shared parameters—supporting reads (SR), variant allele frequency (VAF), and mapping quality (MAPQ)—were consistently tuned across Nanomonsv, Severus, and Savana. Full parameter settings and command lines are provided in the Supplementary Material for transparency and reproducibility.

### 2.3 Ensemble integration of SV calls

The four SV files derived from ONT and those from PacBio were subsequently integrated independently. Merging was performed with SURVIVOR (version 1.0.7) using the official recommended settings: a maximum breakpoint distance = ±1000 bp, required agreement on SV type and strand orientation, and a minimum SV length threshold of 30 bp (Jeffares et al., 2017). The only difference between ensemble strategies was the minimum number of supporting tools, which was set to 2 or 3. As a result, four ensemble files were generated in total, named ensemble-ONT-2, ensemble-ONT-3, ensemble-PacBio-2, and ensemble-PacBio-3, and these merged call sets were subsequently used in the benchmarking and evaluation analyses.

### 2.4 Benchmarking and performance evaluation

Performance evaluation was conducted against the NIST-HG008 draft somatic SV benchmark (version 0.3, released on 21 February 2025), hereafter referred to as NIST-HG008 (McDaniel et al., 2025). Truvari (version 5.3.0) was used to compare individual SV call sets and ensemble callsets against the NIST-HG008 (English et al., 2022). The following parameters were applied: *-sizemax = -1* (disabling the maximum variant size limit), *-passonly*, and *-pick multi*. The GIAB GRCh38 reference genome was used, along with the BED file *GRCh38_HG008-T-V0.3_somatic-stvar.draftbenchmark.bed* to restrict evaluation to benchmark regions; only calls within these regions were counted. All other parameters were left at default values. Parameter choices generally followed GIAB recommendations, except that we removed the 50-kb size constraint by setting *-sizemax* to retain large SVs that would otherwise be filtered out by the default of Truvari.

In addition, functional characterization was performed as a key component of the analysis. SVs were annotated using AnnotSV (v3.0.9) (Geoffroy et al., 2018) with respect to the GRCh38 reference genome to obtain gene-level annotations. The resulting gene sets were intersected with the Cancer Gene Census from COSMIC (Tate et al., 2019) to identify known driver genes, and with the OncoKB actionable gene list (Suehnholz et al., 2024) to identify clinically relevant genes.

### 2.5 Methodological comparison and downstream annotation of ensemble SV callsets

To benchmark the ensemble strategy, we reproduced a previously published multi-tool integration workflow, in which eight SV callers were executed with a standardized minimum SV length threshold of 50 bp (Aydin et al., 2025). Candidate somatic SVs were derived through merging and subtraction-based filtering, retaining variants supported by at least six tools (Comb6). The resulting Comb6 callset was evaluated against the NIST-HG008 using Truvari under the same assessment criteria as our framework to ensure direct and consistent comparison.

For downstream characterization, insertion sequences were extracted from the ensemble callsets and annotated using RepeatMasker (v4.2.2) (Smit and Hubley, 2025) to determine repetitive element composition. The functional impact of all SVs was assessed using AnnotSV under the same annotation pipeline as described above. Together, these analyses provide integrated structural and functional evidence supporting the ensemble SV dataset.

## 3 Results

### 3.1 Somatic SV detection on ONT dataset

**FIGURE 1:**
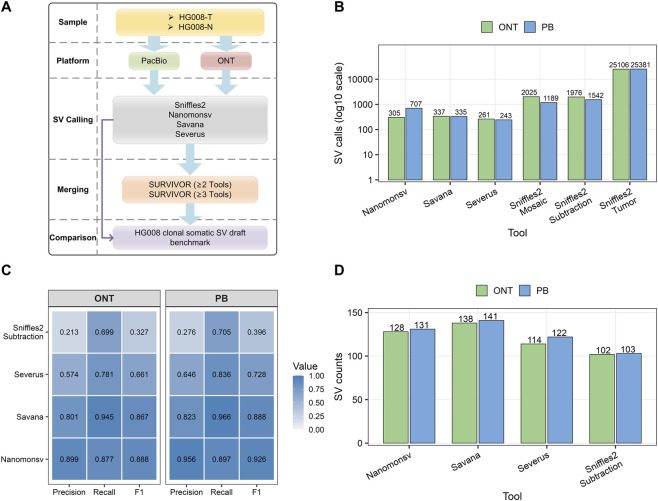
Comparison of somatic SV callers across sequencing platforms. **(A)** Schematic overview of the benchmarking workflow, including paired HG008 tumor and normal samples, ONT and PacBio sequencing, SV detection with multiple callers, and comparison against the HG008 clonal somatic SV draft benchmark. **(B)** Total numbers of somatic SVs detected by each caller across ONT and PacBio datasets, shown on a log10 scale. **(C)** Performance comparison of SV callers across ONT and PB datasets in terms of precision, recall, and f1 score, evaluated against the NIST-HG008 benchmark set. **(D)** Numbers of detected somatic SVs that overlap the NIST-HG008 somatic SV benchmark set for each caller across platforms.

The ONT dataset included a tumor sample (HG008-T) sequenced at an apparent diploid coverage of 63× and a matched control (HG008-N) at 41× coverage, with both datasets aligned to the GRCh38-GIABv3 reference genome using minimap2 and achieving high mapping rates. Four SV detection tools were applied to call somatic SVs from these data (Figure 1A). Among them, Sniffles2 offered the simplest workflow and the fastest processing speed and can operate without a matched control. In contrast, Savana and Severus incorporate additional preprocessing steps, including SNP calling, phasing, and haplotagging, which result in substantially longer runtimes—4.8 h and 7.3 h, respectively—compared with Sniffles2 at 16.1 min and Nanomonsv at 2 h, underscoring pronounced differences in computational efficiency. In terms of detection outcomes, Sniffles2 was evaluated under three strategies. Tumor-only mode yielded over 25,000 variants, mosaic mode detected more than 2,000 SVs, and the tumor–normal subtraction approach identified 1,976 somatic variants—the highest among all methods. In contrast, the other three tools each detected approximately 200–300 somatic SV calls (Figure 1B; Supplementary Table S1).

We employed the Truvari tool to compare the results from each SV caller against NIST-HG008. NIST-HG008 was created by integrating results from multiple sequencing platforms, including ONT, PacBio, and Illumina, called by seven somatic SV detection tools. All candidate SVs were subjected to manual curation and validation, resulting in a high-confidence reference set comprising 149 somatic SVs. In the evaluation against NIST-HG008, Savana identified 138 somatic SVs and achieved the highest recall (0.945), indicating strong sensitivity (Figures 1C,D). Nanomonsv and Severus detected 128 and 114 SVs, respectively. Among all tools, Nanomonsv achieved the highest precision (0.899) and f1 score (0.888), reflecting a more balanced performance between precision and recall, while Severus showed moderate performance. It is worth noting that the tumor-only mode of Sniffles2 was excluded from benchmarking, as it does not distinguish somatic from germline variants and is therefore unsuitable for somatic SV analysis. Only the subtraction and mosaic modes were evaluated. The subtraction strategy improved precision and f1 score but remained inferior to other tools, while the mosaic mode failed to detect any benchmark SVs.

### 3.2 Performance characteristics of Sniffles2 modes

Sniffles2 was unable to detect any somatic SVs in the NIST-HG008 dataset under its mosaic mode, primarily because the algorithm is tailored for low-frequency variants. The mosaic mode aims to identify SVs with VAFs of 5%–20%, and more than 2,000 events detected under the ONT mosaic mode fell within this range (Smolka et al., 2024). By contrast, the somatic SVs in NIST-HG008 are largely clonal events with higher VAFs, and all true SVs detected in the default mode exceeded the mosaic threshold. This indicates that the mosaic setting is unsuitable for somatic SV detection in clonal samples, as it tends to miss medium-to high-frequency signals.

To further validate this point, we increased the*–mosaic-af-max* threshold in mosaic mode to 50%, 60%, and 70%. In the ONT data, the number of detected SVs increased from approximately 7,000 to over 10,000 as the threshold was relaxed; however, only 15, 24, and 27 variants overlapped with the benchmark set, remaining substantially lower than those detected by other tools (Supplementary Figure S1). This suggests that even with relaxed thresholds, the single-tumor mosaic mode remains prone to under-detection in tumors dominated by clonal somatic variants. Collectively, these results emphasize that the effectiveness of the mosaic mode is highly context-dependent and varies with the clonal architecture of the tumor.

### 3.3 Somatic SV detection on PacBio dataset

The same analytical pipeline was applied to the PacBio dataset, in which the tumor sample was aligned with pbmm2 at 106× coverage and the matched control at 68× depth, both achieving near-complete mapping rates to the GRCh38-GIABv3 reference genome. Similarly, Sniffles2 produced the highest SV call counts across all modes, with 25,381 variants in tumor-only mode, whereas mosaic and subtraction modes yielded markedly fewer calls (Figure 1B; Supplementary Table S1). Likely due to the difference in sequencing depth, Nanomonsv detected significantly more SVs than in the ONT dataset, approximately 700 in total. For the other two tools, the number of somatic SVs detected was relatively consistent between the two datasets. These SVs were subsequently compared with the NIST-HG008 benchmark, where likewise no overlap was detected for Sniffles2 in the mosaic setting. A similar trend was observed in the PacBio dataset: Savana achieved the highest recall, while Nanomonsv showed the best overall performance, and Severus exhibited intermediate performance. In contrast, Sniffles2 showed consistently lower accuracy in the subtraction mode (Figures 1C,D).

### 3.4 Comprehensive characteristics of somatic SVs detected by different callers

**FIGURE 2:**
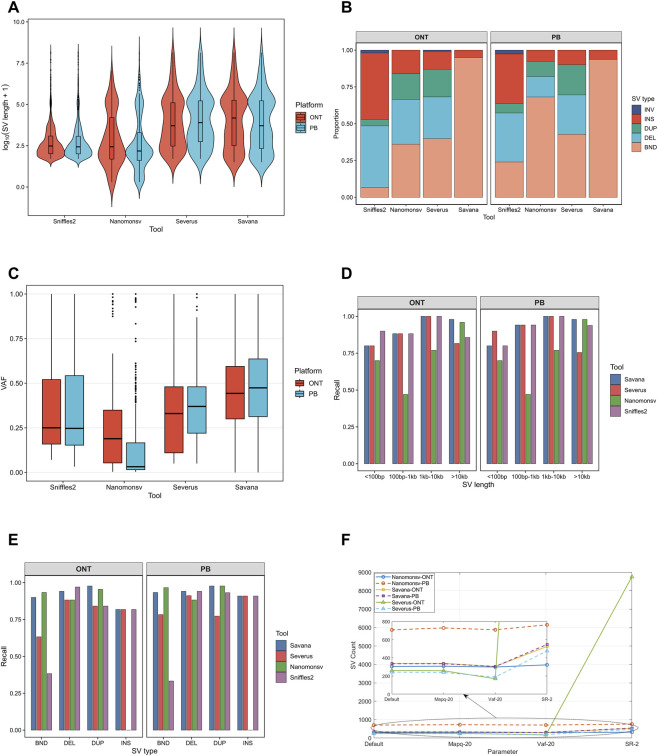
A comprehensive comparison of somatic SV detection across different callers (including Sniffles2, Nanomonsv, Severus, and Savana) and sequencing platforms. Notably, the Sniffles2 results were obtained using a tumor–normal subtraction strategy to filter calls, rather than an intrinsic mode of the algorithm. **(A)** SV length distributions reveal distinct caller-specific preferences across ONT and PB datasets. **(B)** SV type compositions highlight substantial variability in SV categories detected by different callers. **(C)** VAF distributions demonstrate differences in sensitivity to low- and high-frequency variants. **(D,E)** Recall performance stratified by SV length and type, respectively, showing differential strengths of callers across variant classes. **(F)** Impact of parameter settings on SV detection, illustrating the robustness and sensitivity of different callers under varying filtering conditions.

Building on the benchmark-based evaluation, we further characterized somatic SVs across length, type, and VAF to reveal caller-specific detection preferences. We first examined the overall distributions of SVs across these three dimensions for all four callers. Sniffles2 (tumor–normal subtraction) and Nanomonsv were biased toward shorter SVs, although Nanomonsv exhibited a longer tail toward large variants. In contrast, Severus and Savana showed similar distributions, both centered on medium-to-large SVs (Figure 2A). Savana was dominated by BND events across both platforms, while Sniffles2 primarily detected deletions and insertions. Severus showed relatively balanced type distributions, whereas Nanomonsv exhibited platform-dependent patterns, appearing more balanced on ONT but enriched for BND events on PB (Figure 2B). VAF patterns indicated that Savana favored high-VAF variants, Nanomonsv was more sensitive to low-VAF events, and Severus and Sniffles2 showed intermediate or dispersed distributions (Figure 2C).

Due to the absence of VAF annotations and the enrichment of high-clonality events in the NIST-HG008, VAF was excluded from recall-based comparisons. Sniffles2 and Severus favored shorter variants, Nanomonsv favored large SVs (>10 kb), whereas Savana showed more consistent performance across the length spectrum (Figure 2D). At the SV type level, Nanomonsv and Savana performed better in BND and duplication detection, although Nanomonsv showed low sensitivity for insertion. Severus exhibited balanced performance, whereas Sniffles2 underperformed for complex SVs but retained advantages in insertion and deletion detection (Figure 2E). Overall, consistent trends were observed between the two sequencing platforms across all analyses, with only minor differences.

Moreover, given that functional relevance represents a critical dimension for evaluating SV callers, we performed gene-level annotation of detected SVs and assessed their overlap with the COSMIC and OncoKB gene lists. As shown in Supplementary Table S2, Sniffles2 identified the largest number of SVs across both sequencing platforms, leading to the highest gene coverage and the greatest overlap with these databases. In contrast, Nanomonsv, Severus, and Savana generated more conservative call sets, with correspondingly fewer annotated genes and database overlaps. Despite these differences in SV counts, all tools showed strong concordance in identifying key cancer-related genes. Notably, the canonical tumor suppressor *CDKN2A*, the actionable kinase *CHEK1*, and the epigenetic regulator *SUZ12* were consistently detected across all callers, whereas the well-known oncogenic driver *KRAS* was identified by all tools except Savana. These findings indicate that even relatively compact call sets can retain core variants of biological and clinical significance.

### 3.5 Effects of parameter configuration on tool performance

We conducted systematic parameter optimization and sensitivity analyses, harmonizing three key metrics including VAF, MAPQ, and SR across tools, and applied a stringent yet widely accepted strategy with VAF ≥20%, MAPQ ≥20, and supporting reads ≥2 to prioritize robust somatic events, reduce mapping artifacts, and achieve a balanced trade-off between sensitivity and specificity (Sedlazeck et al., 2018; Cameron et al., 2019; Chen et al., 2020; Jin et al., 2022). Notably, Sniffles2 was excluded from this analysis as its subtraction-based workflow differs fundamentally from the tumor–normal joint modeling strategies used by the other tools, rendering key parameters such as VAF and SR not directly comparable. Accordingly, parameter sensitivity analyses were restricted to Nanomonsv, Savana, and Severus. The results revealed marked differences in how the tools respond to parameter changes. Nanomonsv showed the most stable SV counts across all parameter settings on both platforms (Figure 2F; Supplementary Figure S2). Savana was mainly sensitive to the SR threshold, with lower SR increasing SV counts but reducing the f1 score. In contrast, Severus was sensitive to both VAF and SR; raising VAF improved its f1 score, whereas lowering SR to 2 led to a dramatic increase in SV calls, exceeding 8,700 events in the ONT dataset and indicating elevated noise. Overall, these findings indicate that Nanomonsv exhibits the most stable performance under parameter perturbations, while the default settings of all three tools strike a favorable balance between sensitivity and specificity.

### 3.6 Evaluation of multi-tool ensemble strategy

**FIGURE 3:**
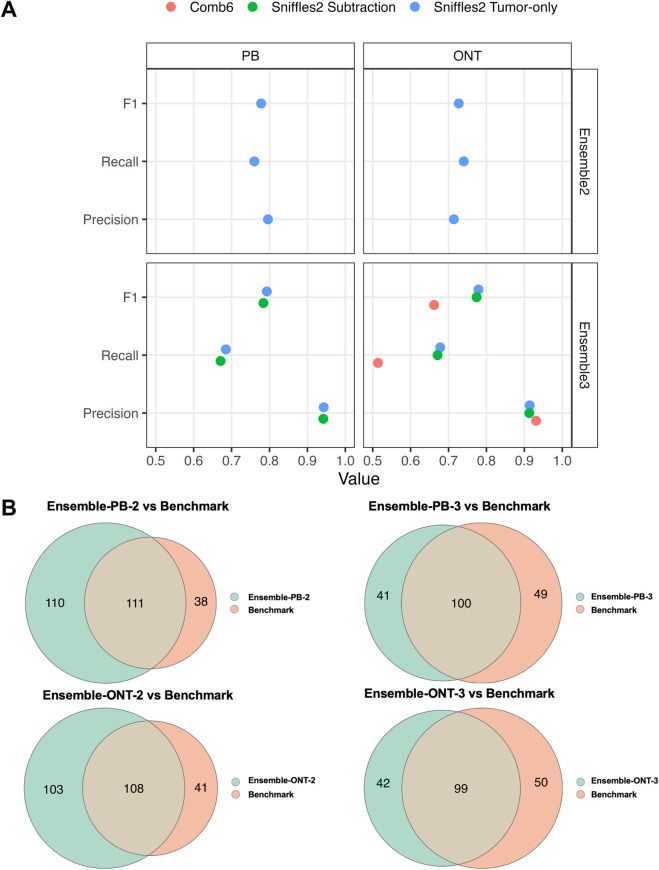
Comparison of different SV ensemble methods. **(A)** Performance comparison of ensemble2 and ensemble3 across ONT and PB datasets under different Sniffles2 calling strategies (tumor-only and tumor–normal subtraction), with additional comparison to Comb6, evaluated by precision, recall, and f1 score. **(B)** Overlap between ensemble callsets and the NIST-HG008 benchmark across ONT and PacBio datasets.

Motivated by tool-specific SV biases, we adopted a prevailing multi-tool ensemble strategy and evaluated its performance for somatic SV identification across both PacBio and ONT datasets. Building on prior work (Martín et al., 2024), we first examined the impact of tuning Sniffles2 calling modes on detection performance. Specifically, we compared tumor-only and subtraction modes within the ensemble-3 framework, constructed using SURVIVOR by integrating SVs supported by at least three of the four callers across both datasets. The tumor-only mode consistently yielded higher f1 scores across platforms, driven by improved recall at comparable precision, while also offering superior computational efficiency (Figure 3A). The results establish the tumor-only mode as a more practical approach for ensemble-based SV detection.

Accordingly, we further compared the performance of ensemble-2 and ensemble-3 under the tumor-only setting to assess how different support thresholds (requiring support from at least two or three callers) influence the trade-off between sensitivity and reliability. For PacBio, ensemble-PB-2 included 221 SVs with 111 overlapping NIST-HG008 and an f1 score of 0.778, whereas ensemble-PB-3 contained 141 SVs with 100 overlaps and a higher f1 score of 0.793 (Figures 3A,B-upper). Consistent results were obtained for ONT, where ensemble-ONT-3 outperformed ensemble-ONT-2 (Figures 3A,B-lower). These findings support the use of ensemble-3 as a more reliable strategy for somatic SV detection.

### 3.7 Comparative evaluation and functional characterization of ensemble SV callsets

To rigorously assess the performance of ensemble-3, we benchmarked it against a previously published multi-tool integration framework that aggregates eight SV callers to construct a composite somatic callset. Notably, only Severus was explicitly developed for somatic SV detection, whereas the remaining tools were originally designed for germline analysis, rendering the framework conceptually distinct from the somatic-oriented ensemble-3 strategy. In that approach, only variants supported by at least six of the eight callers were retained (Comb6). As Nanovar requires allele-specific tags that are unavailable in the PacBio dataset, the comparison was therefore conducted between Comb6 and ensemble-ONT-3 under identical benchmarking criteria. Ensemble-ONT-3 overlapped with 99 benchmark SVs, compared with 75 for Comb6, achieving an f1 score of 0.779 *versus* 0.662 (Figure 3A). Collectively, these findings demonstrate that ensemble-3 delivers greater robustness and practical utility for routine somatic SV analysis.

Ensemble-3 callsets were annotated with RepeatMasker and AnnotSV to integrate repeat composition and functional impact. Insertion sequences, as the only SV type introducing novel DNA, were specifically analyzed by RepeatMasker. Insertions from ensemble-ONT-3 and ensemble-PB-3 exhibited highly concordant repeat profiles across platforms and were predominantly enriched for LINE elements, consistent with LINE-1–mediated retrotransposition (Supplementary Table S3). Many events also contained SINE elements such as AluJb together with poly-A/T low-complexity sequences, often forming chimeric multi-fragment architectures. In aggregate, both ONT and PacBio data support cancer-related transposition-driven somatic insertions accompanied by repeat-mediated rearrangements, reinforcing the biological validity of the ensemble-3 callset.

Systematic functional annotation of ensemble-ONT-3 and ensemble-PB-3 using AnnotSV demonstrated high completeness and interpretability, with all SVs receiving valid annotations. The ONT dataset yielded 747 non-redundant genes, whereas the PacBio dataset identified 329 non-redundant genes, with 313 genes shared between the two platforms, indicating strong cross-platform concordance and complementary gene coverage (see Supplementary Material). Enrichment analysis revealed significant involvement in cancer-related pathways, particularly DNA damage repair and oncogenic signaling, with representative genes such as *KRAS*, *CDKN2A*, and *ERCC1*. Taken together, these findings substantiate the tumor relevance of the detected SVs and highlight the cross-platform robustness of the ensemble-3 framework.

## 4 Discussion

To address the longstanding challenge of accurate somatic SV detection in cancer genomes, we systematically benchmarked four somatic-oriented callers using the HG008 long-read dataset and developed a streamlined ensemble-3 integration framework. Across both ONT and PacBio platforms, our results reveal substantial differences in detection performance, algorithmic preferences, and parameter sensitivity, reflecting fundamental trade-offs inherent in their modeling frameworks.

Overall, Sniffles2 offers a simple workflow and high computational efficiency, making it suitable for rapid screening scenarios. However, applying a tumor–normal subtraction strategy to derive somatic calls from Sniffles2 still leads to inferior overall performance compared with other tools. Moreover, the mosaic mode, designed for low-VAF variants, performs poorly in clonal tumor settings, highlighting its strong context dependency. In contrast, Nanomonsv shows the most balanced performance, achieving the highest precision and f1 score while maintaining a strong balance between sensitivity and specificity. It also exhibits strong robustness to parameter perturbations, although its performance in insertion detection remains relatively limited. Savana is characterized by high sensitivity, consistently achieving the highest recall in benchmark comparisons, particularly for high-VAF and breakpoint-driven BND events. However, its performance is strongly dependent on parameter settings, especially SR thresholds, and is prone to increased noise under relaxed filtering conditions. Severus shows relatively balanced performance across SV types and sizes, with strength in detecting complex and large-scale variants, but shows high sensitivity to VAF and SR thresholds, leading to substantial inflation of variant calls under permissive settings and reduced stability.

Despite these differences in call set size and performance metrics, all tools show strong concordance in identifying key cancer-associated genes, suggesting that core biological signals are largely preserved. However, this apparent consistency is primarily driven by high-confidence clonal events, whereas substantial discrepancies remain for more complex or low-frequency variants. These findings indicate that no single tool can comprehensively capture the somatic SV landscape, and that integrating complementary detection strategies is essential for achieving accurate and reliable results in practical research and clinical applications (Liu et al., 2024).

Building upon these observations, we implemented a multi-tool integration workflow and compared different consensus strategies. Ensemble-3 improves accuracy and substantially reduces false positives, while also outperforming the previously published Comb6 framework in terms of benchmark overlap and f1 score under identical evaluation settings. These findings suggest that a task-oriented, streamlined integration strategy is more effective than simply increasing the number of tools. Moreover, functional annotation further supports biological relevance of the ensemble-3, with insertions predominantly driven by LINE-1 retrotransposition and affected genes significantly enriched in cancer-related pathways, including DNA repair and oncogenic signaling. As a complementary approach, Ensemble-3 provides a simple and practical option for somatic SV integration. However, its effectiveness may vary depending on the application scenario and should be interpreted with reference to the benchmarking results.

Overall, this study provides a rigorous evaluation of current somatic SV detection tools and establishes a multi-tool integration framework for cancer genomics and clinical research. Extensive validation in additional cell lines will be key to consolidating its effectiveness and paving the way for its broad adoption in cancer genomics and precision medicine.

## Data Availability

The original contributions presented in the study are included in the article/Supplementary Material, further inquiries can be directed to the corresponding authors.
