# Supplementary material for: Benchmarking major somatic structural variant callers on the HG008 genome
Source: Front Genet. 2026 May 12;17:1732039. doi: 10.3389/fgene.2026.1732039 (PMC13200840; doi:10.3389/fgene.2026.1732039)
Supplement: Supplementary file 1 [file DataSheet1.pdf]

frontiers

Supplementary Material

1 Supplementary Note 1. Supplementary Methods

1.1 Data sources and reference genome

We obtained four paired tumor–normal long-read sequencing datasets generated on Oxford Nanopore Technologies (ONT) and PacBio platforms from the 42basepairs portal, which provides public access to GIAB somatic datasets via both the GIAB FTP site and the GIAB S3 bucket (https://42basepairs.com/browse/web/giab/data_somatic/HG008/; https://42basepairs.com/browse/s3/giab/data_somatic/HG008). These datasets were generated as part of the Genome in a Bottle (GIAB) Consortium and were used as the primary benchmarking resource for somatic structural variant (SV) detection in this study.

Nanopore sequencing was performed on the PromethION 48 platform using R10.4.1 flow cells. Basecalling was carried out using dorado (version 0.3.4), which completed successfully without reported errors. Reads were subsequently aligned to the GIAB-customized GRCh38 reference genome (GRCh38-GIABv3) using Minimap2 (version 2.26-r1175). Raw read quality was assessed using pycoQC, while alignment-level quality metrics were evaluated using Cramino (version 0.13.0). Genome-wide coverage was calculated using the Snakemake wrapper of mosdepth (version 3.3.6).

For the HG008-T tumor sample, approximately 6.67 million reads were confidently mapped (80.19%), yielding a read N50 of 35.5 kb and an apparent diploid coverage of approximately 63×. For the matched normal sample (HG008-N), 9,591,713 reads were mapped (77.34%), with a read N50 of 24,460 bp and an average genome-wide coverage of approximately 41×. These metrics collectively indicate robust sequencing performance and reliable mapping quality for the ONT datasets.

PacBio long-read sequencing data were generated following the standard Revio HiFi SMRTbell library preparation and sequencing protocol. Reads were aligned to the same GRCh38-GIABv3 reference genome using pbmm2 (version 1.12.0), followed by alignment quality assessment with Cramino (version 0.14.5) and coverage estimation with mosdepth (version 0.3.6). For the HG008-T tumor sample, 12,974,130 reads were mapped (99.9%), achieving a read N50 of 18,132 bp and an apparent diploid coverage of approximately 106×. For the matched normal sample, 13,268,351 reads were mapped (99.99%), with a read N50 of 16,658 bp and an average coverage of approximately 68×, reflecting high sequencing quality and near-complete mapping efficiency.

The GRCh38-GIABv3 reference genome used throughout this study is derived from GRCh38 but excludes alternative contigs and masks low-complexity and decoy regions, including *MAP2K3*, *KMT2C*, and *KCNJ18*. To ensure analytical consistency and comparability across tools and platforms, all downstream SV analyses were conducted using this reference genome.

1.2 Somatic SV calling pipeline

Somatic SVs were detected directly from the downloaded, pre-aligned BAM files using four state-of-the-art long-read SV callers: Sniffles2, Nanomonsv, Savana, and Severus. All tools were primarily

executed in tumor–normal paired mode to ensure consistency with the study design. To enable a fair comparison, Sniffles2 was evaluated using three strategies: (i) tumor-only mode, (ii) tumor-only mosaic mode enabled with the --mosaic option, and (iii) a tumor–normal subtraction approach for somatic SV detection. In the subtraction strategy, SVs were independently called in tumor and normal samples using Sniffles2, and tumor-specific variants were subsequently identified using SURVIVOR.

Unless otherwise specified, default parameters were used for all tools, including minimum supporting reads and minimum SV length thresholds. For Savana, platform-specific models were explicitly specified using the *--ont* or *--pb* option, as recommended by the developers.

Prior to somatic SV calling, both Savana and Severus strongly recommend the use of phased and haplotagged alignment files as input, as these tools leverage haplotype information to improve breakpoint resolution, distinguish allelic origins, and reduce false positives in complex tumor genomes characterized by copy number alterations, allelic imbalance, and structural rearrangements. Accordingly, we strictly followed the official recommended workflows for each tool and explicitly implemented three sequential steps: SNV calling, phasing, and haplotagging, to generate high-quality input data and maximize SV detection performance.

For Savana, we first performed germline SNP calling on the matched normal sample using Clair3 (v1.0.10) to obtain reliable heterozygous variants as the basis for phasing. We then applied WhatsHap (v2.6) to generate a phased VCF file from these SNPs. Finally, WhatsHap was used again to haplotag sequencing reads by haplotype, producing the haplotagged BAM file required by Savana as its final input.

Similarly, Clair3 was applied to the normal BAM file for SNP calling and phasing as part of the Severus workflow. Subsequently, WhatsHap leveraged the phased file to haplotag both the normal and tumor BAM files, allowing Severus to reliably distinguish somatic events from germline background signals.

1.3 Parameter sensitivity analysis

To assess the robustness of somatic SV detection and evaluate the influence of key parameters on tool performance, we conducted systematic parameter sensitivity analyses. For Sniffles2 mosaic mode, the maximum allele frequency threshold (*--mosaic-af-max*) was tested at 0.5, 0.6, and 0.7.

For Nanomonsv, Severus, and Savana, we focused on three universally shared and most influential parameters in somatic SV detection: supporting reads (SR), variant allele frequency (VAF), and mapping quality (MAPQ). Specifically, we tuned *-min_tumor_variant_read_num*, *-min_tumor_VAF*, and *-var_read_min_mapq* for Nanomonsv; *-min-support*, *-vaf-thr*, and *-min-mapq* for Severus; and *-min_support*, *-min_af*, and *-mapq* for Savana. This harmonized parameter tuning strategy enables a principled and fair comparison across tools by aligning core evidence thresholds related to read support, clonal signal strength, and alignment confidence.

1.4 Ensemble integration of SV calls

SV callsets generated by the four tools were integrated separately for ONT and PacBio datasets. Merging was performed using SURVIVOR with the officially recommended configuration, including

a maximum breakpoint distance of ±1000 bp, required agreement on SV type and strand orientation, and a minimum SV length threshold of 30 bp. The only difference between ensemble strategies was the minimum number of supporting tools required for an SV to be retained, which was set to either two or three. This resulted in four ensemble callsets: Ensemble-ONT-2, Ensemble-ONT-3, Ensemble-PacBio-2, and Ensemble-PacBio-3. These ensemble callsets were used in all subsequent benchmarking and evaluation analyses.

1.5 Benchmarking and performance evaluation

Performance evaluation was conducted against the NIST-HG008 draft somatic SV benchmark (NIST-HG008). Truvari was used to compare individual SV callsets and ensemble callsets against the benchmark truth set. Benchmarking was performed using the following parameters: *--sizemax=-1* to disable the maximum SV size limit, *--passonly* to restrict evaluation to high-confidence calls, and *--pick multi* to accommodate representational variability in long-read SV calls. The GIAB GRCh38 reference genome was used in conjunction with the BED file *GRCh38_HG008-T-V0.3_somatic-stvar.draftbenchmark.bed* to restrict evaluation to benchmarked regions; only SVs falling within these regions were counted. All other parameters were left at default values. Parameter choices largely followed GIAB and NIST recommendations.

1.6 Manual curation, methodological comparison, and downstream annotation

Variants classified as false positives in the benchmarking analysis were manually inspected using IGV. Original tumor–normal BAM files were loaded, and each candidate locus was visually inspected under GRCh38 coordinates. Variants showing strong SV signals in tumor samples but absent in matched normals were considered likely true somatic events missed by the benchmark set.

To further contextualize the performance of the proposed ensemble framework, we compared it with a previously published benchmark approach and strictly reproduced the original publicly available code and workflow. Specifically, eight widely used SV callers, including Sniffles2, cuteSV (version 2.1.3), Delly (version 1.7.2), DeBreak (version 1.0.2), Dysgu (version 1.7.0), NanoVar (version 1.8.3), SVIM (version 2.0.0), and Severus, were applied separately to tumor and matched normal samples for independent SV detection, with a standardized minimum SV length threshold of 50 bp. Candidate somatic SVs were then identified through VCF merging and subtraction-based filtering. Among these tools, Severus accepts tumor and normal data simultaneously and directly outputs somatic SV calls, whereas the remaining callers were run independently on tumor and normal samples. The original study systematically evaluated combinations ranging from comb2 to comb8 and reported that comb6 achieved the highest f1 score. Therefore, we adopted the same criterion and retained only SVs supported by at least six of the eight tools as the Comb6 reference set. Finally, using the same evaluation framework as in our study, we compared the Comb6 callset against the NIST-HG008 benchmark using Truvari to ensure consistent and comparable performance assessment.

For downstream annotation analyses, insertion sequences were first extracted separately from the ensemble callsets. RepeatMasker was then applied to these insertion sequences using the hg38 reference genome to systematically characterize their repetitive element composition. Subsequently, functional annotation of the ensemble sets was performed using AnnotSV, also based on the hg38 reference genome. AnnotSV was executed with default parameters to assess the potential impact of each SV on genes, regulatory elements. This annotation framework provides a systematic

characterization spanning sequence features to functional impacts, thereby establishing a dual evidence framework at both structural and functional levels for the ensemble dataset.

2 Supplementary Note 2. Full Command Lines and Parameters

Sniffles2

sniffles -i tumor.bam -v Sniffles2_tumor_output.vcf

sniffles -i nromal.bam -v Sniffles2_normal_output.vcf

SURVIVOR merge sample_files 1000 1 1 1 0 30 sample_merged.vcf

sniffles --input tumor.bam --vcf Sniffles2_output.vcf --mosaic

sniffles --input tumor.bam --vcf Sniffles2_somatic_50.vcf --mosaic --mosaic-af-max 0.5

sniffles --input tumor.bam --vcf Sniffles2_somatic_50.vcf --mosaic --mosaic-af-max 0.6

sniffles --input tumor.bam --vcf Sniffles2_somatic_50.vcf --mosaic --mosaic-af-max 0.7

NanoMonSV

nanomonsv parse normal.bam nanomonsv/008_normal

nanomonsv parse tumor.bam nanomonsv/008_tumor

nanomonsv get nanomonsv/008_tumor tumor.bam reference.fasta --control_prefix nanomonsv/008_normal --control_bam normal.bam --processes 8 --use_racon

nanomonsv get nanomonsv/008_tumor tumor.bam reference.fasta --control_prefix nanomonsv/008_normal --control_bam normal.bam --processes 8 --use_racon --var_read_min_mapq 20

nanomonsv get nanomonsv/008_tumor tumor.bam reference.fasta --control_prefix nanomonsv/008_normal --control_bam normal.bam --processes 8 --use_racon --min_tumor_VAF 0.2

nanomonsv get nanomonsv/008_tumor tumor.bam reference.fasta --control_prefix nanomonsv/008_normal --control_bam normal.bam --processes 8 --use_racon --min_tumor_variant_read_num 2

SAVANA

MODEL_NAME="r1041_e82_400bps_sup_v520" or "hifi_revio"

run_clair3.sh --bam_fn=normal.bam --ref_fn= reference.fasta --threads=32 --platform="ont" or "hifi" --model_path="/path/${MODEL_NAME}" --output=clair3_out --enable_phasing --longphase_for_phasing

whatshap phase --ignore-read-groups -o phased_008_output.vcf.gz --reference= reference.fasta clair3_out/merge_output.vcf.gz normal.bam

whatshap haplotag --ignore-read-groups -o phased_tumour.bam --reference reference.fasta phased_008_output.vcf.gz tumor.bam && samtools index phased_tumour.bam

whatshap haplotag --ignore-read-groups -o phased_normal.bam --reference reference.fasta phased_008_output.vcf.gz normal.bam && samtools index phased_normal.bam

savana --tumour phased_tumour.bam --normal phased_normal.bam --outdir savana_outdir –ref reference.fasta --contigs contigs.txt --pb or –ont

savana --tumour phased_tumour.bam --normal phased_normal.bam --outdir savana_outdir –ref reference.fasta --contigs contigs.txt --pb or --ont --mapq 20

savana --tumour phased_tumour.bam --normal phased_normal.bam --outdir savana_outdir –ref reference.fasta --contigs contigs.txt --pb or --ont --min_af 0.2

savana --tumour phased_tumour.bam --normal phased_normal.bam --outdir savana_outdir –ref reference.fasta --contigs contigs.txt --pb or --ont --min_support 2

Severus

MODEL_NAME="r1041_e82_400bps_sup_v520" or "hifi_revio"

run_clair3.sh --bam_fn=normal.bam --ref_fn= reference.fasta --threads=32 --platform="ont" or "hifi" --model_path="/path/${MODEL_NAME}" --output=clair3_out --enable_phasing --longphase_for_phasing

whatshap haplotag --reference reference.fasta clair3_out/phased_merge_output.vcf.gz normal.bam -o normal.haplotagged.bam --ignore-read-groups --tag-supplementary --skip-missing-contigs --output-threads=16 && samtools index normal.haplotagged.bam

whatshap haplotag --reference reference.fasta clair3_out/phased_merge_output.vcf.gz tumor.bam -o tumor.haplotagged.bam --ignore-read-groups --tag-supplementary --skip-missing-contigs --output-threads=16 && samtools index tumor.haplotagged.bam

severus --target-bam tumor.haplotagged.bam --control-bam normal.haplotagged.bam --out-dir severus_out -t 32 --phasing-vcf clair3_out/phased_merge_output.vcf.gz --vntr-bed human_GRCh38_no_alt_analysis_set.trf.bed

severus --target-bam tumor.haplotagged.bam --control-bam normal.haplotagged.bam --out-dir severus_out -t 32 --phasing-vcf clair3_out/phased_merge_output.vcf.gz --vntr-bed human_GRCh38_no_alt_analysis_set.trf.bed --min-mapq 20

severus --target-bam tumor.haplotagged.bam --control-bam normal.haplotagged.bam --out-dir severus_out -t 32 --phasing-vcf clair3_out/phased_merge_output.vcf.gz --vntr-bed human_GRCh38_no_alt_analysis_set.trf.bed --vaf-thr 0.2

severus --target-bam tumor.haplotagged.bam --control-bam normal.haplotagged.bam --out-dir severus_out -t 32 --phasing-vcf clair3_out/phased_merge_output.vcf.gz --vntr-bed human_GRCh38_no_alt_analysis_set.trf.bed --min-support 2

SURVIVOR

SURVIVOR merge sample_files 1000 2 1 1 0 30 sample_merged.vcf

SURVIVOR merge sample_files 1000 3 1 1 0 30 sample_merged.vcf

---

Truvari

truvari bench -b GRCh38_HG008-T-V0.3_somatic-stvar_PASS.draftbenchmark.vcf.gz -c merged.vcf.gz -- reference.fasta --includebed GRCh38_HG008-T-V0.3_somatic-stvar.draftbenchmark.bed --passonly --pick multi --sizemax -1 -o truvari

---

3 Supplementary Note 3. Gene-level functional annotation and biological summary

Functional annotation of ensemble-supported SVs identified a substantial set of genes recurrently affected across both ONT and PacBio platforms. Among the cross-platform consensus set, the affected genes span diverse functional categories, including transcriptional regulation, chromatin organization, signal transduction, cell-cycle control, DNA damage response, and cellular metabolism. Notably, several genes with established relevance to cancer biology and genome stability were identified, such as *KRAS*, *CDKN2A*, *RBBP8*, *NHEJ1*, *SKP2*, *HELLS*, *RAC1*, and *MAP3K7*, highlighting the biological plausibility of the detected somatic SVs.

In addition to protein-coding genes, the ensemble callsets also affected a substantial number of long non-coding RNAs such as *LINC00333*, *LINC00550*, *LINC01239*, *SNHG14*, *SNHG17* and microRNAs such as *MIR1343*, *MIR3611*, *MIR548M*, suggesting potential regulatory consequences beyond direct gene disruption. Several genes involved in cell adhesion including *CD44*, *NECTIN2*, *PCDH9*, immune signaling containing *SLAMF1*, *SLAMF6*, *CD48*, and neuronal or developmental pathways such as *SALL4*, *LMO2*, *RSPO2*, *GDNF* were also recurrently affected. Importantly, the presence of these genes across both sequencing platforms indicates strong cross-platform concordance and supports the robustness of the ensemble-based detection strategy.

4 Supplementary Figures and Tables

4.1 Supplementary Tables

Supplementary Table 1. Summary of structural variant (SV) calls detected by different tools on ONT and PB datasets.

Tool	Platform	Total SVs	BND	DEL	DUP	INS	INV
Severus	ONT	261	104	74	49	32	2
Savana	ONT	337	320	-	-	17	-
Nanomonsv	ONT	305	110	93	53	49	-
Sniffles2-mosaic	ONT	2025	19	1054	18	933	1
Sniffles2-subtraction	ONT	1976	134	825	87	892	38
Sniffles2	ONT	25106	247	10701	171	13854	133
Severus	PacBio	243	104	65	50	24	0
Savana	PacBio	335	314	-	-	21	-
Nanomonsv	PacBio	707	482	98	73	54	-
Sniffles2-mosaic	PacBio	1189	16	521	5	646	1
Sniffles2-subtraction	PacBio	1542	370	513	101	518	40
Sniffles2	PacBio	25381	860	10055	225	14075	166

Note: DEL = deletion; DUP = duplication; INS = insertion; INV = inversion; BND = breakend. Counts are based on high-confidence SV calls.

Supplementary Table 2. Summary of gene-level annotations and cancer-related database overlaps for SV calls detected by different tools across ONT and PacBio datasets.

Tool	Platform	Annotated Genes	COSMIC Overlap	OncoKB Overlap
Severus	ONT	1512	47	10
Savana	ONT	129	9	3
Nanomonsv	ONT	830	28	6
Sniffles2-Subtraction	ONT	4094	117	29
Severus	PacBio	687	23	7
Savana	PacBio	126	7	3
Nanomonsv	PacBio	2268	63	18
Sniffles2-Subtraction	PacBio	4433	126	30

Note: Annotated genes were obtained using AnnotSV. COSMIC and OncoKB overlaps indicate the number of genes intersecting with the COSMIC cancer gene census and the OncoKB database, respectively. Sniffles2 results are based on the tumor–normal subtraction mode.

Supplementary Table 3. Repeat composition of insertion sequences identified by Ensemble-3 SV callsets.

Repeat class / family	PacBio	ONT	Representative subfamilies
LINE/L1	High (dominant)	High (dominant)	L1HS, L1P1, L1MA10, L1M1
SINE/Alu	Moderate	Moderate	AluJb, FRAM
Simple_repeat	Frequent	Frequent	(A)n, (T)n
LINE/L2	Low	Low	L2d2

Note: Counts are based on RepeatMasker annotation of insertion sequences derived from ensemble-supported SVs.

4.2 Supplementary Figures

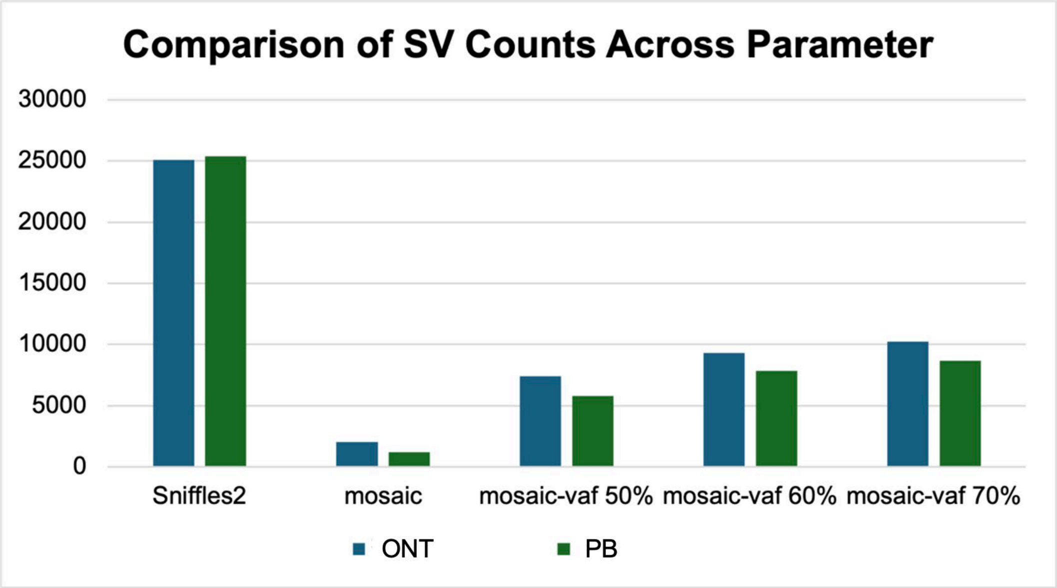

Supplementary Figure S1. Comparison of SV detection counts under different parameter settings in ONT and PacBio datasets. The total number of SVs identified by Sniffles2 (default mode) and mosaic mode under varying VAF thresholds (50%, 60%, and 70%) are shown for ONT and PB data.

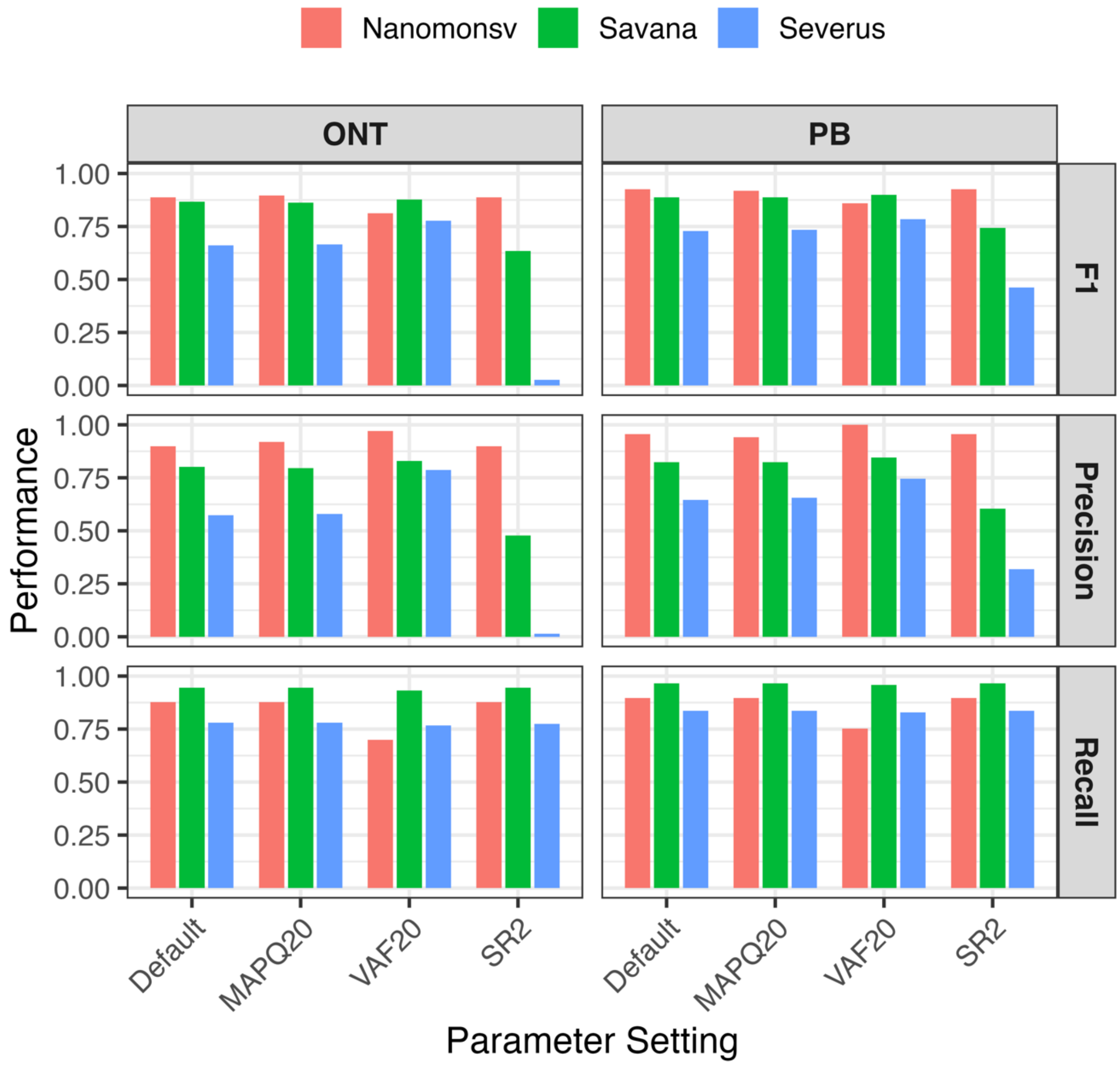

Supplementary Figure S2. Performance comparison of SV callers under different parameter settings across ONT and PacBio datasets. Precision, recall, and F1-score of Nanomonsv, Savana, and Severus are evaluated under default and modified parameter thresholds (MAPQ $\geqslant$ 20, VAF $\geqslant$ 20%, and supporting reads $\geqslant$ 2), highlighting the sensitivity of each method to parameter variations across ONT and PacBio (PB) datasets.
